# Foot Gesture Recognition Using High-Compression Radar Signature Image and Deep Learning

**DOI:** 10.3390/s21113937

**Published:** 2021-06-07

**Authors:** Seungeon Song, Bongseok Kim, Sangdong Kim, Jonghun Lee

**Affiliations:** 1 Division of Automotive Technology, Daegu Gyeongbuk Institute of Science and Technology (DGIST), Daegu 42988, Korea; sesong@dgist.ac.kr (S.S.); remnant@dgist.ac.kr (B.K.); kimsd728@dgist.ac.kr (S.K.); 2 Department of Interdisciplinary Engineering, Daegu Gyeongbuk Institute of Science and Technology (DGIST), Daegu 42988, Korea

**Keywords:** Doppler radar, CNN, foot gesture, SVD, STFT, gesture recognition, AlexNet, deep learning

## Abstract

Recently, Doppler radar-based foot gesture recognition has attracted attention as a hands-free tool. Doppler radar-based recognition for various foot gestures is still very challenging. So far, no studies have yet dealt deeply with recognition of various foot gestures based on Doppler radar and a deep learning model. In this paper, we propose a method of foot gesture recognition using a new high-compression radar signature image and deep learning. By means of a deep learning AlexNet model, a new high-compression radar signature is created by extracting dominant features via Singular Value Decomposition (SVD) processing; four different foot gestures including kicking, swinging, sliding, and tapping are recognized. Instead of using an original radar signature, the proposed method improves the memory efficiency required for deep learning training by using a high-compression radar signature. Original and reconstructed radar images with high compression values of 90%, 95%, and 99% were applied for the deep learning AlexNet model. As experimental results, movements of all four different foot gestures and of a rolling baseball were recognized with an accuracy of approximately 98.64%. In the future, due to the radar’s inherent robustness to the surrounding environment, this foot gesture recognition sensor using Doppler radar and deep learning will be widely useful in future automotive and smart home industry fields.

## 1. Introduction

In recent years, gesture recognition has been playing an important role in opening new ways of human and machine interaction in a wide variety of applications such as wearable devices [1,2,3], smart phones [4], autonomous vehicles [5,6,7], and health care [8].

Existing sensors for a gesture recognition of movements of hands and arms include ultrasound [9,10,11,12], camera based vision [13,14,15,16,17], and radar [18,19,20,21,22,23,24,25,26,27,28]. Ultrasonic sensors have the advantage of relatively low price, but they have short detection distance. Camera based image sensors are a very common gesture recognition approach that use various popular CNN (Convolution Neural Network)-based deep learning models [13,14,15,16,17]. However, because camera based sensors are strongly dependent on surrounding environment factors like lighting, dust, and so on, it is necessary to design sensors very precisely for out-of-vehicle or outdoor use. Radar has the advantage of being strong against noise signals such as those from moisture, dust, and vibration. In addition, radar sensors are suitable for human motion recognition because they have excellent sensitivity and Doppler resolution to changes of moving objects as well as strong immunity to ambient noise.

Among various types of radar sensor, Doppler radar is a good candidate for gesture recognition because it has high sensitivity to human movement. Based on machine-learning and deep learning techniques involving the time-frequency Doppler spectrum, Doppler radar-based gesture recognition has been intensively studied for static and dynamic hand gestures [21,22,24,25] and arm gestures [23,26]. In conventional supervised machine-learning, well-defined extracted features are essential. Typical machine learning methods are the k-nearest neighbor (KNN), support vector machine (SVM), and random forest methods [29,30,31,32,33]. However, the recognition performance is strongly dependent on the predefined features. On the other hand, deep learning approaches based on multi-layer networks such as the convolutional neural network (CNN) are promising for overcoming such feature selection problems without advance need for feature sets. GoogleNet, AlexNet, VGGNet, ResNet, and DenseNet are good examples of deep learning models [34,35,36,37,38,39,40].

Among various methods of gesture recognition, foot gesture recognition is a very useful tool because it allows simple command and control when the hand cannot be freely used. Existing sensors use simple solutions by means of motion detection. In conventional sensors, unexpected door opening may occur due to the unwanted movement of small animals or objects around the sensor. To overcome this problem, the function has been improved in a way that recognizes the user’s foot movement or intention after user authentication through an additional external device such as a smart key fob or smart phone. Nevertheless, the problem of unpredictable door opening remains because it is difficult to grasp the user’s exact intention or foot gesture. In order to solve this misrecognition problem in a more sophisticated smart opening system, it is necessary to recognize foot gestures only with foot motions without a external device. 

Conventional foot gesture recognition is performed using a dual channel surface electromyography (sEMG) wearable band, which has been proposed as a hands-free controller for entertainment applications [41]. In automotive applications, one method involves predicting foot movements toward pedals based on capacitive proximity sensing and the hidden Markov model for robust driver foot tracking and recognition [42,43]. Another method involves detecting only simple kick gestures using Doppler radar and a machine-leaning model as a kick-activation system [27,28]. Existing foot gesture solutions have certain limitations such as inconvenience of contact sensing or confined working zones indoors or inside vehicles.

To overcome these limitations, Doppler radar-based foot gesture recognition has recently attracted attention as a smart trunk opener for smart vehicles and as a smart door opener for smart home applications [44,45,46]. For example, it is very useful to open the trunk of a car or automatically open and close a house front door using foot motion in situations in which hands are unavailable or uncomfortable to use. Unfortunately, so far, Doppler radar-based recognition of various foot gestures is still very challenging.

To the best of our knowledge, no studies have yet deeply dealt with recognition of various foot gestures based on Doppler radar and deep learning. Therefore, in this paper, we propose such a Doppler system for foot gesture recognitions. In addition, through the proposed method, we evaluate the recognition performance of four different foot gestures and examine impacts on recognition of foot gestures of sudden moving objects such as rolling objects, passing animals, and so on. To increase the recognition performance, the deep learning-based recognition requires a vast number of radar images for deep learning training [47,48].

To overcome the requirement of large memory, a newly extracted high-compression radar signature image is utilized for deep learning based foot gesture recognition. The proposed technique creates a new high-compression radar signature by extracting a dominant feature via SVD (Singular Value Decomposition) processing and recognizes four different foot gestures via a deep learning model, without degradation of gesture recognition. Instead of a high-resolution original radar signature, the proposed method improves the required memory efficiency for deep learning training by using a high-compression radar signature.

This paper presents a new method for effectively selecting valid radar data through high memory space efficiency and data visualization in the configuration of a large-capacity model training data set required for gesture recognition using a radar signature and deep learning. In other words, by converting the radar STFT signature transparently mapped to Gray or RGB image instead of dealing with the raw beat signal of the radar output signal, the visibility of the radar signature can be clearly checked, so that valid radar data for various gesture targets can be easily selected and thus, a large radar data set is built. On the other hand, when the STFT radar signature is converted to an image, some signals of the radar signature are lost due to the quantization error. To minimize this quantization error, the STFT radar signature was converted to an RGB image instead of a gray image.

Foot gestures of interest include kicking, swinging, sliding, and tapping; also considered is a moving object, namely a baseball that rolls near the radar sensor and passes it. This object can have a significant impact on foot gesture recognition because the four different foot gestures and the movement of the rolling baseball have very similar movement patterns.

The remainder of this paper is organized as follows. Section 2 presents the signal model of the Doppler radar used for foot gesture recognition. Section 3 presents a new method using a high-compression radar signature image and deep learning model with singular value decomposition scheme for foot gesture recognition. Section 4 details various experiments and the obtained original and reconstructed radar signature images for different foot gestures. In Section 5, experimental recognition results of four different foot gestures are provided. Finally, conclusions are given in Section 6.

## 2. Signal Model

This section describes the system model of the Doppler radar for foot gesture recognition. Doppler radar can estimate Doppler signatures corresponding to unique foot motions. Particularly, Doppler signals include micro-Doppler frequency components due to movements of non-rigid feet and ankles and main Doppler shift components caused by translational motions of the body.

In a typical Doppler radar, a transmitted (TX) signal is reflected from a foot gesture. The reflected signal is changed into a beat signal at the receiver (RX). The sinusoidal signal’s frequency is sensitive to small movements of the foot. A system model of CW (continuous wave) radar is considered for a single human subject. The CW TX signal is transformed into a continuous wave signal by $s_{\mathrm{tx}}\left(t\right)=\exp\left(j2\pi f_{c}t\right),$ where *f_c_* is the center frequency.

For the Doppler estimation of a set of L rigid and non-rigid targets with different radial velocities {$v_{m}\left(t\right),$ for m=1, …, L}, foot movements will change the targets’ Doppler frequency shifts. These frequencies correspond to {$f_{m}\left(t\right)=2f_{c}v_{m}\left(t\right)/C,$ for m=1,…,L} where *C* is the velocity of light. The beat signal of a single frame for a *CW* radar is obtained by mixing the transmitted and received signals. The beat signal *x*$\left(t\right)$ at a single frame is represented by

(1) $$x\left(t\right)=\underset{I\text{-}\mathrm{channel}}{\underbrace{\overset{L}{\underset{m=1}{\sum }}x_{m,I}\left(t\right)}}+\underset{Q\text{-}\mathrm{channel}}{j\underbrace{\overset{L}{\underset{m=1}{\sum }}x_{m,Q}\left(t\right)}}=\overset{L}{\underset{m=1}{\sum }}\left[\underset{x_{m,I}\left(t\right)}{\underbrace{a_{m}\cos(2\pi f_{m}t)}}+j\underset{x_{m,Q}\left(t\right)}{\underbrace{a_{m}\sin(2\pi f_{m}t)}}\right]$$

where $x_{m,I}\left(t\right)$ and $x_{m,Q}\left(t\right)$ are the I-channel and Q-channel components, respectively, of a beat signal consisting of L human’s body motion components due to a foot gesture. $a_{m}$ and $f_{m}$ are the amplitude and the micro-Doppler frequency components, respectively. After passing through an ADC (analog-to-digital converter) with sampling rate of $f_{s}=1/T_{s}$, the digitized beat signal at a single frame is denoted by $X\left[n\right]$ and obtained by substituting $t=nT_{s}$ into $\;X\left(t\right)$, that is, $X\left(t\right)=X\left(nT_{s}\right)$, where $T_{s}$ is the sampling time interval.

By transforming the digitized beat signal $X\left[n\right]$ via the FFT (Fast Fourier transform), an STFT (Short-Time Fourier Transform) spectrogram $X\left(k,f\right)$ can be determined as

(2) $$X\left(k,f\right)=\overset{M-1}{\underset{n=0}{\sum }}X\left[n\right]\omega \left[k-n\right]e^{-\frac{j2\pi fn}{N}}$$

where $\omega \left[n\right]$ is a window function and M and N are the number of samples per frame and the point of FFT, respectively.

Equations (1) and (2) ensure that beat signals have different amplitude and frequency components for each foot movement. By extracting these unique radar signatures in the time and frequency domains in a beat signal, different foot movements can be distinguished.

## 3. New Foot Gesture Recognition Method Using High-Compression Radar Signature Image and Deep Learning Model

**Figure 1:**
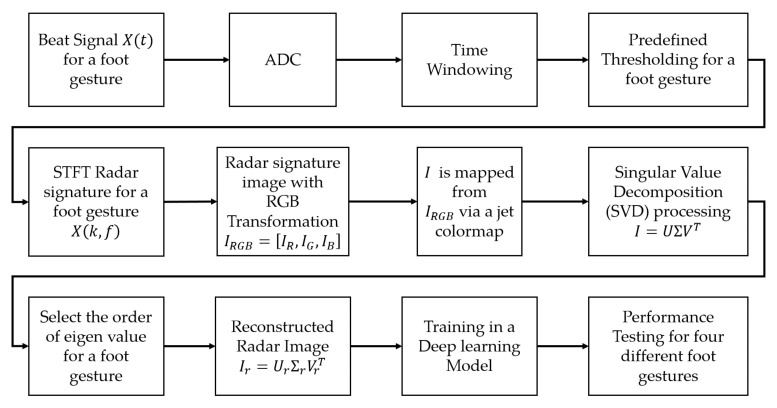
New recognition method using new radar signature image and deep learning via singular value decomposition for foot gesture.

Figure 1 shows a new method of using a high-compression radar signature image and deep learning for foot gesture recognition.

The newly proposed foot gesture recognition method is largely composed of a six-step procedure. The entire procedure includes (1) acquisition of beat signal for each foot gesture, (2) calculation of STFT radar spectrogram, (3) conversion of STFT radar signature RGB image, (4) selection of order of eigenvalue, (5) reconstruction of new radar signature image, and (6) deep learning processing for foot gesture recognition.

In the first step, a beat signal $x\left(t\right)$ for different foot gestures is obtained using Equation (1). In the second step, the STFT spectrogram $X\left(k,f\right)$ is extracted from the received beat signal using Equation (2). Then, only the Doppler frequency of interest corresponding to the foot gesture is selected through appropriate frequency filtering in the spectrogram.

In the third step, a radar signature RGB image I is obtained by using RGB transformation and color mapping to convert the STFT spectrogram. To construct radar data sets for deep learning training as easily as possible, visualization of radar data is quite useful. In other words, instead of storing the beat signal (raw data) to select valid data for various foot gestures, the visibility of the radar signal can be easily checked by means of the STFT radar signature RGB image, so it is easy to pick up the valid data corresponding to the short movement. On the other hand, the radar signature signal suffers from the quantization error caused by the conversion of the STFT radar signature to the RGB image.

To provide a constant-resolution analysis in both time and frequency domains for robust classification, the reconstructed radar RGB image shall satisfy shift-invariance property in time and frequency domains because the STFT radar signature satisfies this shift-invariance property. To meet this shift-invariance property, during the compression, the STFT radar signature is converted into an RGB image with a single array proportional to the magnitude through a colormap.

In the fourth step, an SVD (Singular Value Decomposition) technique is performed from the radar signature image *I* and the order of its eigenvalues for feature extraction is selected. Singular value decomposition (SVD) and principal component analysis (PCA) are two popular eigenvalue methods used to reduce a high-dimensional data set into fewer dimensions while retaining important information. SVD is very similar to PCA because of a data decomposition approach for feature extraction of a signal. SVD is based on a single datapoint while PCA require a vast amount of data. In other words, PCA cannot use a single data to pick up features and cannot provide details of the dominant features. Moreover, different foot gestures are usually hidden in certain specific times and frequencies, so using SVD to extract the dominant features for a single radar signal allows for the collection of more comprehensive information than using PCA.

To extract dominant feature information of a foot movement feature from the original radar image *I*, the SVD technique is utilized. For convenience, the value of the RGB image, expressed as an array of three numbers, is mapped to an array of a single number via color mapping. Here, a jet colormap is used. The final converted RGB image has a value of one dimension, not three dimensions as obtained via jet colormap. Therefore, the value of the converted radar RGB image I corresponds to the magnitude of the STFT radar signature. Then, the transformed image is scaled to the size of p by $q\;\left(p>q\right)$ for input into the deep learning model. Generally speaking, computational complexity uses the number of Floating-point Operation(FLOP). Assuming that we use radar images of size p × q (p = q = 227) and truncated SVD, the SVD processing requires computational complexity of *O*($3p^{3}+p^{3}+p$) FLOP.

Singular value decomposition for radar signature RGB image is expressed as

(3) $$I=U\Sigma V^{T}$$

where *U* and *V* are the left and right matrix, respectively. The columns of orthogonal matrices *U* and *V* are eigenvectors, and Σ = diag $\left[\sigma_{1},\sigma_{2},\ldots \sigma_{r},\ldots \sigma_{q}\right]$ is the eigen matrix and diag [·] denotes a diagonal matrix operator. That is, *U* and *V*, and Σ, are expressed in the following matrix form:

(4) $$U=[u_{1}\;u_{2}\;\cdots \;u_{r}\;\cdots \;u_{p}],$$

(5) $$\Sigma =[\begin{array}{cccccc} \sigma_{1} & 0 & & \ldots & & 0\\ 0 & \sigma_{2} & & \ldots & & 0\\ & & \ddots & & & \\ \vdots & \vdots & & \sigma_{r} & & \vdots \\ & & & & \ddots & \\ 0 & 0 & & \ldots & & \sigma_{q}\\ 0 & 0 & & \ldots & & 0\\ \vdots & \vdots & & & & \vdots \\ 0 & 0 & & \ldots & & 0 \end{array}],$$

(6) $$V=[v_{1}\;v_{2}\;\cdots \;v_{r}\;\cdots \;v_{p}].$$

By means of the eigenvalues $\sigma_{i},$ and the corresponding eigenvectors $u_{i}$ and $v_{i}$, the radar signature RGB image is rearranged into the following expression:

(7) $$I=\sigma_{1}u_{1}v_{1}^{T}+\sigma_{2}u_{2}v_{2}^{T}+\cdots +\sigma_{r}u_{r}v_{r}^{T}+\cdots +\sigma_{q}u_{q}v_{q}^{T},$$

where $\sigma_{i}$ is the eigenvalue and has a descending order, i.e., $\sigma_{1}>\sigma_{2}>\ldots >\sigma_{r}>\ldots >\sigma_{q}$, and $u_{i}$ and $v_{i}$ are unit orthogonal column vectors corresponding to the eigenvalues. Equation (7) shows that the radar signature RGB image consists of a weighted linear combination of different features.

It is important to determine the eigenvalues and their corresponding eigenvectors so as to include sufficient feature information for each foot movement in the original radar RGB image *I*. By observing the knee points for all foot gestures, which change rapidly, the threshold of the order of the eigenvalue can be well selected. By means of selection of the order, a unique and sufficient feature can be extracted, and the high-compression radar image is simultaneously created in the radar signature image because the order of the eigenvalues can be small relative to the maximum number q. This allows the size of the radar signature image to be dramatically reduced.

When the STFT radar signature is converted into an RGB image, the size of the radar signature is one-dimensional, whereas the RGB image is separated into three dimensions, so the original radar signature can be distorted. To overcome this problem (to match the dimensions of the radar signature and the RGB image), it is converted into an RGB image that can be expressed as a one-dimensional single value mapped through a colormap.

**Figure 2:**
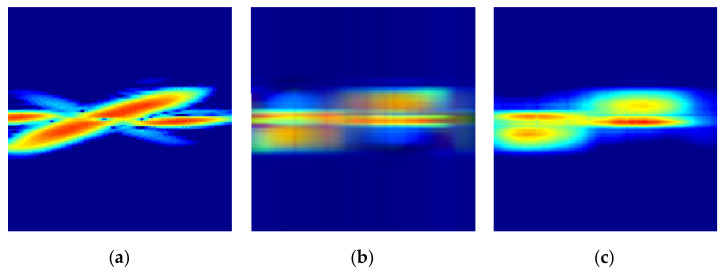
The original and reconstructed STFT radar signatures from general RGB image and RGB image via colormap: (**a**) Original Image; (**b**) Reconstructed image from general RGB image; (**c**) Reconstructed image from RGB image via a colormap.

Figure 2 shows the original radar image, the reconstructed radar image from a RGB image with three dimensional arrays, and the reconstructed radar image from a RGB image with a single dimensional array via a colormap.

In the fifth step, a reconstructed radar signature image with a high-impact feature is generated. Using the selected order *r*, a new radar signature image is reconstructed by the following equation.

(8) $$I_{r}=U_{r}\Sigma_{r}V_{r}^{T}$$

where $U_{r}=[u_{1}u_{2}\cdots u_{r}]$, $\Sigma_{r}=\mathrm{diag}[\sigma_{1}\sigma_{2}\cdots \sigma_{r}]$, and $V_{r}=[v_{1}v_{2}\cdots v_{r}]$; r is relatively small compared to q, r≪q. From Equation (8), a high-compression radar signature images with sufficient features for each foot gesture are newly created.

In the final step, foot gestures are classified by deep learning. The newly proposed radar image was trained and validated for a well-known deep learning model; foot gesture recognition is evaluated through a confusion matrix.

## 4. Experiments and Signature Images for Doppler Radar-Based Foot Gesture Recognition

**Figure 3:**
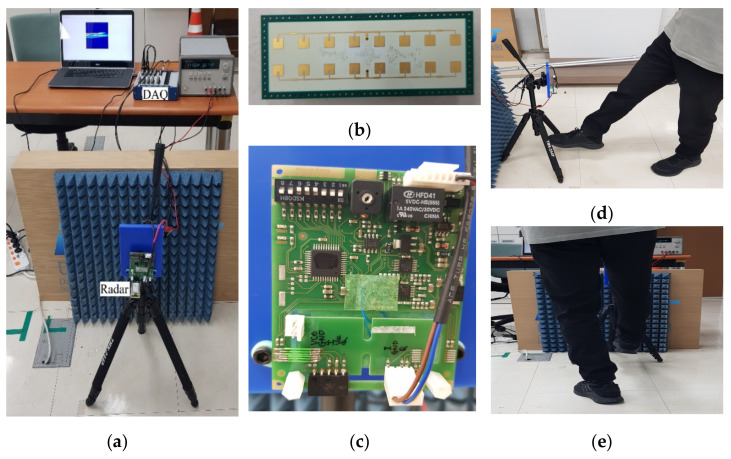
Experimental setup and foot motion scenes for Doppler-radar based foot gesture recognition: (**a**) Experimental setup and data acquisition system; (**b**) Utilized antenna; (**c**) Amplification board for beat signal; (**d**) Side view of foot gesture; (**e**) Front view of same gesture.

Figure 3 shows experimental setup to recognize foot gestures and experimental scenes for obtaining radar signals for different foot gestures. Figure 3a is an experimental setup to collect radar data for foot gestures. Here, the radar sensor used for foot gesture recognition uses a CW modulated waveform. The center frequency is 24 GHz. The maximum detection distance is within about 25 m, with RCS = 0 ${\mathrm{dBm}}^{2}$. Figure 3b,c provide pictures of the utilized antenna and the amplification board of the beat signal, respectively. Here, the horizontal azimuth angle and vertical elevation angle are 80 and 12 degrees, respectively. As can be seen in Figure 3d,e, the radar was installed at a height of about 0.6 m in consideration of a typical deployment location, such as the bumper height of a passenger car or SUV vehicle, or an installation location of a smart door. The radar radiates a transmission signal toward a target. The emitted signal is reflected by movements of the target, that is, foot motion gestures, and returned to the radar. If a received signal is above any predetermined threshold, the received signal is captured via the acquisition system after amplification. Radar STFT signature is transformed by performing a short-time Fourier transform on a PC. Table 1 summarized the used radar parameters for foot gesture recognition.

**Figure 4:**
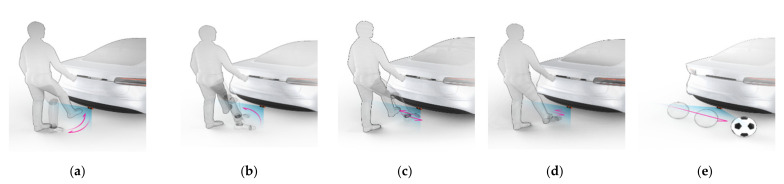
Illustration of different foot gestures used in experiment: (**a**) Kicking; (**b**) Swinging; (**c**) Sliding; (**d**) Tapping; (**e**) Rolling ball.

Figure 4 illustrates different kinds of foot gesture used in this experiment. As can be seen in Figure 4, the four different foot gestures include kicking, swinging, sliding, and tapping; also included is a sudden movement like that of a rolling baseball. Among the different foot movements, kicking extends one foot from the front of the radar in the direction of irradiation and then returns it to the original position; swinging involves one foot moving from a starting a point approximately 45 degrees with respect to the direction of the radiation, to a point approximately 45 degrees along a curved line; sliding involves moving one foot from one side to another in a straight line; tapping involves moving foot up and down, slowly raising the foot in front of the radar and then rapidly bringing it down; the moving object motion is of a baseball rolling near the radar.

The radar signatures are specific and unique and can be distinguished for the different foot gestures. Here, clutter components caused by various stationary obstacles surrounding the radar and DC components due to leaky signals are removed by pre-signal processing before performing a STFT processing. In addition, beat signal is sampled at a sampling rate of 10 KHz for each time window of 409.6 ms. During the STFT processing, time window of 409.6 ms has four sub-time intervals of 102.4 ms. A 10 kHz sampling for 409.6 ms will produce data of 4096 points. The sampled data of 4096 points becomes data size of 1024 × 3073 by doing STFT operation having the parameters such as the point of FFT of 1024 and the size of overlap of 1023. The STFT radar image is finally extracted at a frame rate of 4.9 fps.

To configure the data set for each foot gesture, the STFT radar spectrogram was transformed into an RGB image. The STFT radar spectrogram has a size of 1024 × 3073. Only the frequency shift of a foot gesture in the STFT radar spectrogram is extracted. From various experimental results, Doppler frequencies caused by foot gestures are mainly in the range of −250 Hz to 250 Hz. The Doppler frequency of interest for foot gesture recognition is marginally chosen from 500 Hz to 500 Hz. Therefore, the STFT radar spectrogram of 1024 × 3073 size is converted to an original RGB image of 105 × 3073 size. Therefore, radar spectrum with size of 1024 × 3073 was converted to a 105 × 3073 RGB image, and the converted RGB image is rescaled to an RGB image of 227 × 227, which becomes the input for the deep learning model. Here, to convert the STFT radar spectrogram into an RGB image, the STFT radar spectrogram was transformed into an RGB image using a jet colormap.

**Figure 5:**
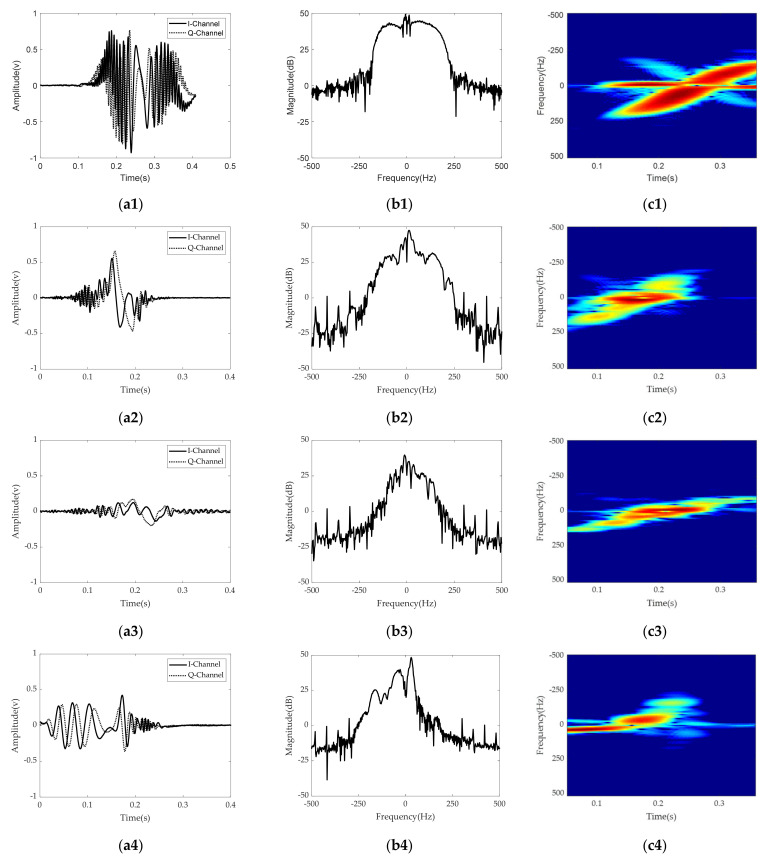

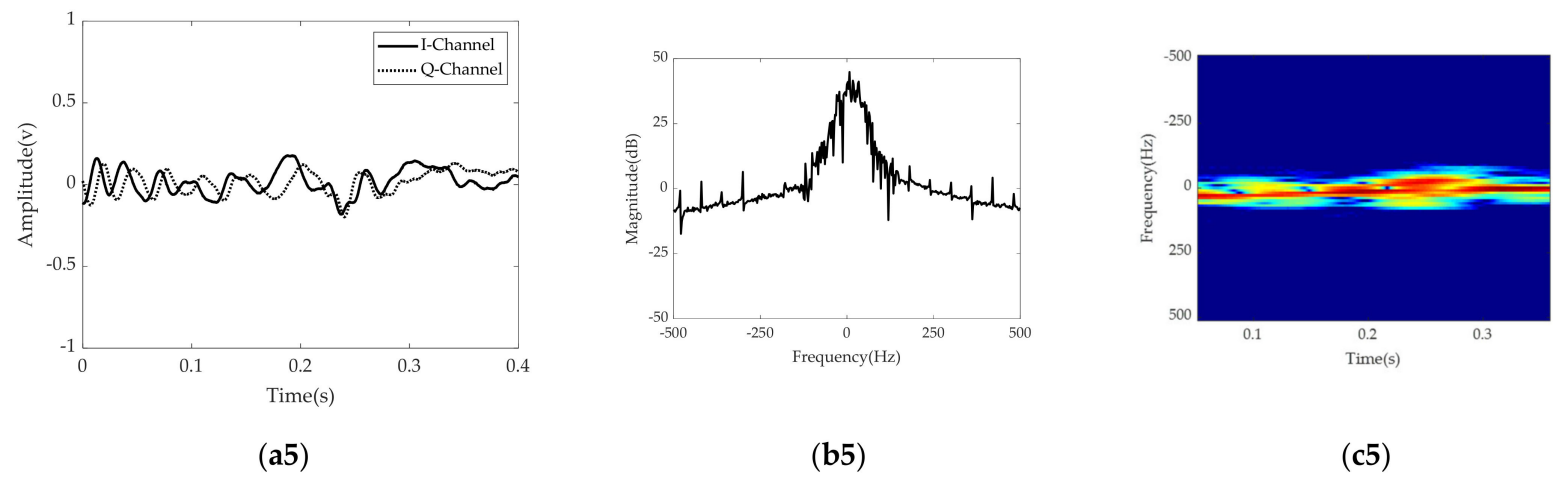
Radar signature for kicking: (**a1**) Time domain; (**b1**) Frequency domain; (**c1**) STFT spectrogram.Radar. signature for swinging: (**a2**) Time domain; (**b2**) Frequency domain; (**c2**) STFT spectrogram.Radar. signature for sliding: (**a3**) Time domain; (**b3**) Frequency domain; (**c3**) STFT spectrogram. Radar signature for tapping: (**a4**) Time domain; (**b4**) Frequency domain; (**c4**) STFT spectrogram. Radar signature for a rolling baseball: (**a5**) Time domain; (**b5**) Frequency domain; (**c5**) STFT spectrogram.

Figure 5 shows radar signatures in the time and frequency domains and the STFT spectrogram for four different foot gestures and one rolling ball. In Figure 5, each row represents a radar signature for a specific recognition target. The first, second, and third columns show radar signals in the time domain, radar signals in the frequency spectrum, and the STFT spectrogram for each target, respectively. The STFT spectrogram is obtained by performing STFT processing with the following Kaiser window function [49,50].

(9) $$\omega \left[n\right]=\frac{I_{0}\left(\beta \sqrt{1-{\left(\frac{n-N/2}{N/2}\right)}^{2}}\right)}{I_{0}\left(\beta \right)},\;\mathrm{for}\;n=\left[0,1,\ldots N-1\right]$$

where $I_{0}\left(\cdot \right)$ is the modified Bessel function of the first kind with an order of zero and β=5. Because the time window of STFT uses the Kaiser function, which is a unit energy window function, the STFT radar signature is a linear and shift-invariant distribution in time and frequency. Because the STFT radar signature is magnitude-wise converted into a RGB image via a colormap, the reconstructed STFT radar image satisfies shift-invariance property in time and frequency domains. Thus, it provides a constant-resolution analysis in both time and frequency domains for robust classification.

**Figure 6:**
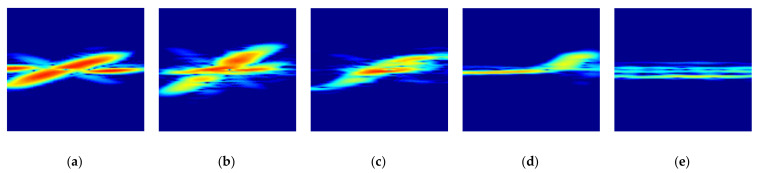
Original STFT radar signature images at a height of about 0.6 m: (**a**) Kicking; (**b**) Swinging; (**c**) Sliding; (**d**) Tapping; (**e**) Rolling baseball.

**Figure 7:**
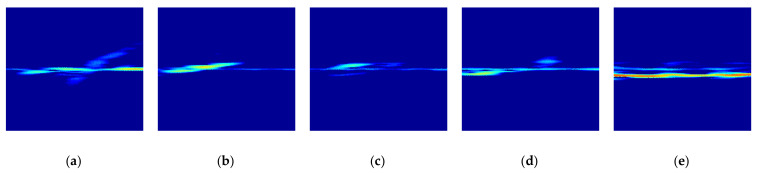
STFT radar signature images at a different height of about 1.5 m: (**a**) Kicking; (**b**) Swinging; (**c**) Sliding; (**d**) Tapping; (**e**) Rolling baseball.

Figure 6 shows the original STFT radar RGB images for the four different foot gestures. As can be seen in Figure 4 and Figure 5, there are clear distinguishing features in the time domain, in the frequency domain, and in the STFT spectrogram for each target. Looking at the symmetry of the time domain and frequency spectrum, kicking and sliding have symmetrical properties, while swinging and tapping have asymmetric characteristics. In addition, from the point of view of bandwidth, kicking, swinging, and tapping have relatively wider bandwidths, while sliding and the rolling baseball exhibit relatively narrower bandwidth due to their different kinetic mechanisms. For example, sliding has a relatively low amplitude and many sidelobes in the frequency domain compared to kicking. Because the movement direction of swinging is almost perpendicular to the direction of radiation, the radial component of the Doppler frequency shift is relatively small. Figure 7 shows original STFT radar signature images for five different gestures measured from the radar installed at another height of about 1.5 m.

Kicking, swinging, and sliding involve motions of the foot approaching and moving away from the radar. Kicking starts with taking the foot off the floor and finishes with a motion of putting the foot back on the floor after fully extending the foot. According to the STFT spectrogram image, the kinetic mechanism allows the sign of the Doppler frequency shift to change from negative to positive and the received signal is stronger than those during swinging or sliding.

In cases of both swinging and sliding, the STFT spectrogram exhibits similar frequency bands but slightly different levels of received power. The strongest received signal is the moment when the foot approaches the radar most closely.

The radar signature in the time domain, the radar signature in the frequency domain, and the STFT spectrum for tapping motion were measured by taking a motion of slowly raising and suddenly lowering the instep. This motion has a unique STFT spectrogram that can be divided into an almost unchangeable and a relatively narrower frequency band in a relatively long interval and a relatively strong and wider frequency band in a very short interval.

The rolling ball has a distinguishable feature in that the STFT spectrogram has several narrow frequency bands due to the ball’s moving speed as well as the ball’s rotational speed during measurement.

**Figure 8:**
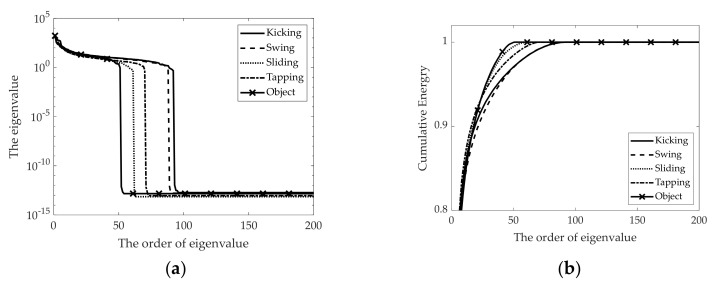
Eigenvalue and cumulative energy in radar signature RGB image for different foot gestures: (**a**) Eigenvalue; (**b**) Cumulative energy.

Figure 8 shows the eigenvalue and cumulative energy of original radar signature RGB images for different foot gestures. From Figure 8, as expected, knee points for all foot gestures that change rapidly are observed; each foot gesture has different knee points, meaning that each foot gesture has a respective domain energy.

**Figure 9:**
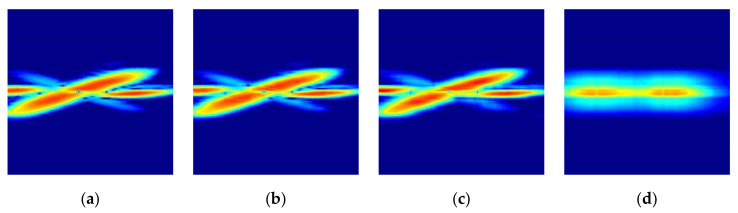
Original and reconstructed radar signature RGB images according to different compression ratios for kicking gesture: (**a**) Original radar image; (**b**) Reconstructed radar image with compression ratio of ϵ = 90%; (**c**) Reconstructed radar image with compression ratio of ϵ = 95%; (**d**) Reconstructed radar image with compression ratio of ϵ = 99%.

Figure 9 shows the original and reconstructed radar signature RGB images according to the different compression ratios for the kicking gesture. Here, the relationship between the order of eigenvalue to be selected and the compression ratio is calculated by

(10) $$r\left(p+q\right)=pq\left(1-\epsilon \right)$$

where r is the order of eigenvalue to be selected and p and q are the dimensions of the original image matrix and ϵ is the compression ratio of the original image to be reconstructed. Figure 9a is the original radar signature RGB image and Figure 9b,d are the reconstructed radar images with different compression ratios ϵ = 90%, 95%, and 99%. Here, the selected order of the eigenvalue r is 11, 6 and 1, respectively.

**Figure 10:**
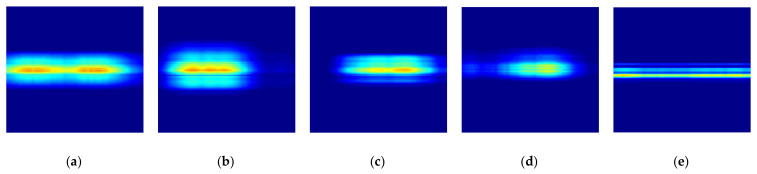
Reconstructed radar signature RGB images according to different foot gestures and movement of moving object, with compression ratio of ϵ = 99%: (**a**) Kicking; (**b**) Swinging; (**c**) Sliding; (**d**) Tapping; (**e**) Rolling ball.

Figure 10 shows the reconstructed radar signature RGB images according to the different foot gestures and the movement of a moving object all with the same compression ratio of ϵ = 99%. Figure 10 corresponds to kicking, swinging, sliding, and tapping movements and the movement of the rolling baseball. Figure 10 provides reconstructed radar signature RGB images for each different foot gesture, which are slightly distinguishable.

## 5. Recognition Results

The original radar signature RGB images for the four different foot gestures and one rolling ball were acquired with 700 images for each target. The total number of original radar images was approximately 3500. To acquire radar images for each foot movement, one woman and four men took part in this experiment. Among the acquired radar images, 600 for each foot gesture were used in a ratio of 90:10 for training and cross-validation for deep learning processing; 100 images for each foot gesture were used to evaluate foot gesture recognition.

To select a learning framework to evaluate the recognition performance of the proposed method, comparative experiments were performed on five different learning models including PCA based SVM, because PCA based SVM method is widely used in the motion classification. In this paper, the learning frameworks considered are GoogleNet, ResNet, VGG, AlexNet, and PCA based SVM [34,39,40,51,52,53]. Comparative recognition performance is summarized in Table 2. In this case, the recognition indicators are recall, precision, F1, and accuracy. Each performance indicator is defined like that recall = TP/(TP + FN), precision = TP/(TP + FP), F1 = 2 (recall × precision)/(recall + precision), and accuracy = (TP + TN)/(TP + FP + FN + TN). Here, TP, TN, FP, and FN are abbreviated as true positive, true negative, false positive, and false negative, respectively. Model training was performed on a PC with Intel (R) core (TM) i7-9800X CPU core, clock speed of 3.80 GHz, RAM of 64 GB, and two nvidia titan GPU computational performance. As can be seen from Table 2, the foot gesture recognition using the proposed high compression method was compared with the others for five different models. Five different learning models have very good performance. In this paper, the AlexNet model was selected among several deep learning models by considering the learning time and recognition performance.

Table 3 shows the accuracy and loss after training and cross-validation based on the AlexNet deep learning model for various radar signature images. With respect to different compression ratios, radar signature images are used to evaluate the recognition performance. Case # 1 is training results using original image; case # 2 uses 90% compressed radar images; cases # 3 and # 4 correspond to radar signature images having 95%, and 99% compression ratios, respectively. Here, the utilized validation frequency is 50; the maximum epochs and size of the mini batches are 30 and 128, respectively. The AlexNet deep learning model was found to have a good accuracy of above 95% even when using radar signature images with compression ratio of 95%.

**Figure 11:**
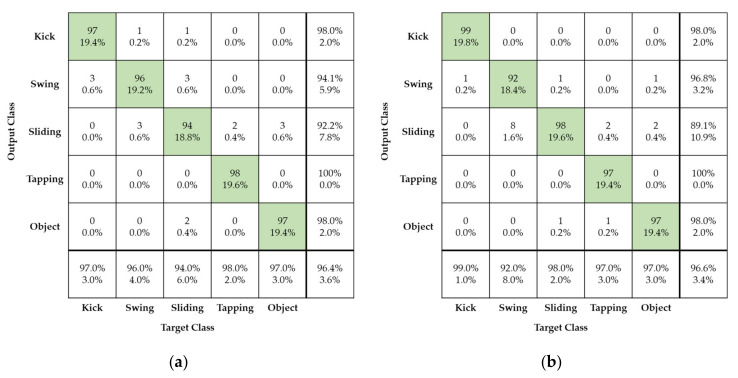

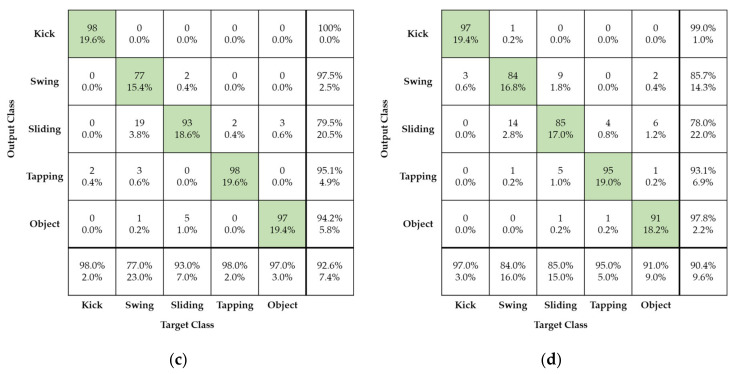
Confusion matrix showing performance of radar sensor’s foot gesture recognition according to original and reconstructed radar images used for both training and testing: (**a**) Original radar image; (**b**) Reconstructed radar image with compression ratio of ϵ = 90%; (**c**) Reconstructed radar image with compression ratio of ϵ = 95%; (**d**) Reconstructed radar image with compression ratio of ϵ = 99%.

Figure 11 shows foot gesture recognition performance obtained using the proposed method in cases of original and reconstructed radar images for both training and test evaluation. Here, four different foot gestures of kicking, swinging, sliding, and tapping are considered as well as the movement of an unpredictable object that may affect foot gesture recognition, a rolling baseball.

As can be seen in Figure 11, the confusion matrix represents the recognition performance of the four different foot gestures using the proposed radar signature image and AlexNet deep learning model. The horizontal and vertical axes in the confusion matrix are the input and output, respectively, of the AlexNet deep learning model.

As can be seen in Figure 11a, all different gestures have good recognition recall of about 96.4% and false recognition rate of about 3.6%. Among the four different foot gestures, tapping has the best recognition, with a recall over 98%. Compared to the other foot gestures, tapping has a short, strong, and clear feature in the radar signature image. On the other hand, sliding, swinging, and the rolling baseball were more easily confused. Sliding and swinging have considerable similarities in kinetic mechanism but a subtle difference that makes it possible to distinguish them. This small difference is caused by the linear and curved movement patterns. Further, sliding and the rolling ball show nearly the same linear movement pattern. There is a subtle difference between the two movements. Sliding involves just linear motion, without up-and-down bouncing, while the rolling ball suffers from up-and-down motion and linear motion simultaneously. This subtle difference makes it possible to clearly distinguish them.

Figure 11b,d show the performance of foot gesture recognition in the case of the proposed high-compression radar images for both training sets. In Figure 11b,c, reconstructed radar images with compression ratios of 90% and 95%, respectively, are used; in Figure 11d, reconstructed radar images with compression ratio of 99% are used.

Figure 11b,d show that the four different foot gestures have nearly identical recognition recall of approximately 96% when comparing case using reconstructed radar image with a compression ratio of 90% and case of using original radar image. The SVD processing extracts the dominant signal components corresponding to large eigenvalues and removes some background noise. So, the accuracy after compression is a little increased due to a denoising effect of the SVD processing.

Figure 11c shows the case of a reconstructed radar image with compression ratio of 95%: the recognition recall is a very high 92%. Among the four different foot gestures, kicking and tapping have especially excellent recall of more than 97%.

In Figure 11d, for a reconstructed radar image with compression rate of 99%, the recognition recall is slightly worse at approximately 90%. Especially, swinging and sliding gestures have recognition recall values of approximately 85%.

To assess the quality of recognition and a more accurate understanding, we depict comparative recognition results for each foot gesture based on reconstructed radar images with different compression ratios graphically and numerically.

**Figure 12:**
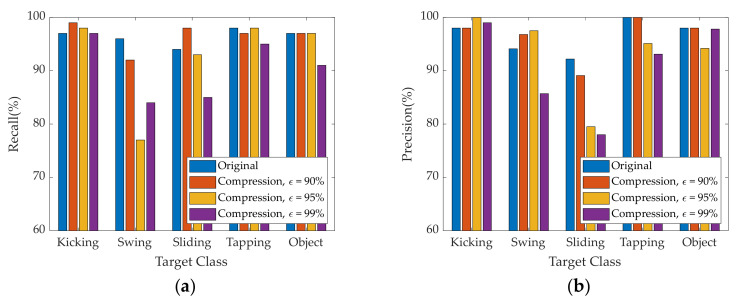
Probabilistic performance of radar sensor’s foot gesture recognition according to original and reconstructed radar images: (**a**) Recall; (**b**) Precision.

Figure 12 shows graphically probabilistic performance of radar sensor’s foot gesture recognition according to original and reconstructed radar images. Table 4 shows more detail of numerically probabilistic performance. Here, recall, precision, and F1 are used as recognition performance for different target gestures.

**Figure 13:**
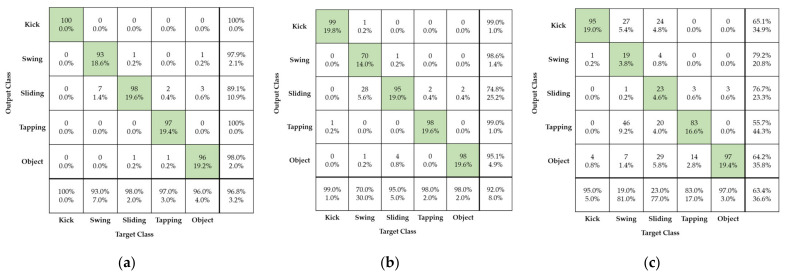
Confusion matrix showing foot gesture recognition performance when compressed and original radar signature images are used as data sets for training and testing, respectively: (**a**) Reconstructed radar of compression ratio of 90% for training and original radar images for testing; (**b**) Reconstructed radar of compression ratio of 95% for training and original radar images for testing; (**c**) Reconstructed radar of compression ratio of 99% for training and original radar images for testing.

Figure 13 shows foot gesture recognition performance when compressed and original radar signature images are used as data sets for training and testing, respectively. Since radar-based deep learning for gesture recognition requires high-volume images for training, it is necessary to use high-compression, low-capacity radar images to improve the memory efficiency. When a highly compressed radar image is used for training and a high-resolution original radar image is used as input of deep learning model, this has a considerable impact on recognition performance of radar-based foot gestures.

In Figure 13a,b, radar images with 90% and 95% compression ratios, respectively, are used for deep learning training. Figure 13a shows recognition recall of foot gestures of approximately 96% when reconstructed radar images with compression ratio of 90% are used for training and original radar image is used for test evaluation.

When radar images with different compression ratios are used for training and testing, the recognition performance of foot gestures is almost the same as in cases using all original radar images and all compressed radar images with compression ratio of 90%, respectively, for training and testing.

Figure 13b shows that when reconstructed radar images with compression ratio of 95% are used for training and original radar images are used for test evaluation, the recognition recall of all foot gestures is approximately 92%. In this case, the recognition performance of foot gestures is almost the same as in the case in which all reconstructed radar images with compression ratio of 95% are used for both training and testing.

Figure 13c shows that when reconstructed radar images with compression ratio of 99% are used for training and original radar images are used as input, the recognition recall of all the foot gestures is approximately 63%. In Figure 13c the recognition recall values for kicking and tapping are 95%, and 83%, respectively. The recognition recall for the rolling ball is approximately 97%. On the other hand, the recognition recall of swinging and sliding were 19% and 23%, respectively, values showing considerable deterioration. From Figure 13, radar-based deep learning for foot gesture recognition can achieve good recognition performance and high memory saving by using as training images radar images at compression ratio of about 90%. Regardless of the use of either compressed or original radar images as input, the recognition recall of foot gestures is over 92%.

**Figure 14:**
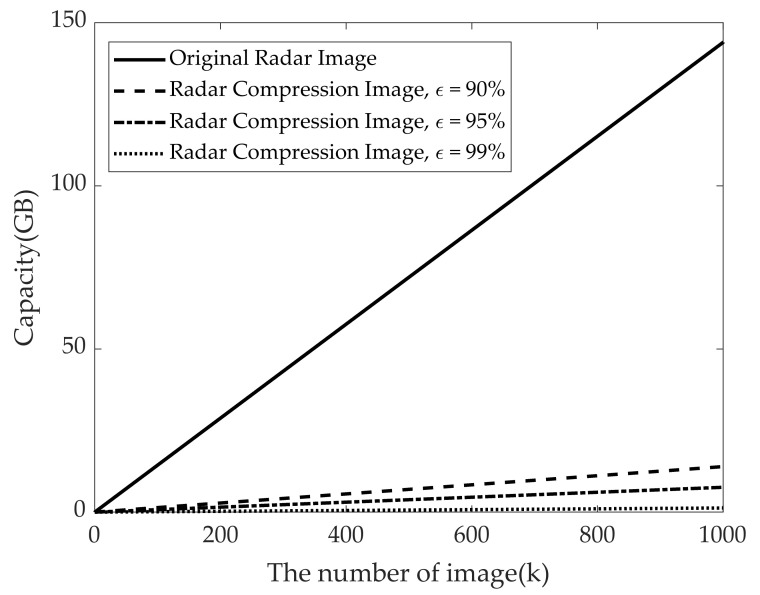
Required memory capacity according to number of training data.

Figure 14 shows the required memory capacity versus the number of training data according to different kinds of radar signature images for foot gesture recognition using radar-based deep learning model. Under the assumption that one pixel image is one byte, the utilized original radar RGB image has approximately 151 Kbytes. As can be seen in Figure 14, compared to using the original radar images, the required memory capacity can be considerably reduced (approximately 19 times) by using radar images with compression ratio of 95%. In other words, if we use a million radar RGB images for deep learning training, memory requirement will be approximately 144 GB. On the other hand, if we use reconstructed radar images with 95% compression ratio, memory of about 8 GB will be required for the same number of images.

## 6. Conclusions

In this paper, we propose a new technique for foot gesture recognition using an SVD based high-compression radar image and a deep learning model. Here, four different foot gestures including kicking, swinging, sliding, and tapping, and the movement of a rolling baseball as an example of something other than a foot movement, were considered. The Doppler radar signature in the time domain, the Doppler radar signature in the frequency domain, and the STFT spectrogram for four different foot gestures were measured and unique radar signatures corresponding to each foot gesture were obtained. In addition, to improve the required memory efficiency, a reconstructed radar signature image with high-compression and a unique dominant feature is created by appropriately selecting high-impact eigenvalues through an SVD processing; the newly proposed radar signature image is used for both training and as input for the deep learning model for foot gesture recognition. Finally, to evaluate the foot gesture recognition performance, AlexNet, a CNN-based deep learning model, was used. The four different foot gestures included kicking, swinging, sliding, and tapping. Original and reconstructed corresponding radar images with high compression values of 90%, 95%, and 99% were used for the AlexNet based deep learning model. As for experimental results, in the case of using a high-compression (95%) radar signature image, all four different foot gestures and the rolling baseball were found to have nearly the same recognition accuracy of approximately 98.64%, similar to that obtained in the original high-resolution radar images.

In the future, due to the radar’s inherent robustness to surrounding environments, foot gesture recognition sensors using Doppler radar and deep learning will be widely useful in future automotive and smart home industry fields such as smart trunk openers, smart door openers, and so on.

## Figures and Tables

**Table 1:** The used radar parameters.

Radar Parameter	Value	Unit
Center frequency	24	GHz
Radar type	CW	-
Sampling rate	10	KHz
Azimuth beam width	80	Degree
Elevation beam width	12	Degree
Maximum detection distance	25 (RCS = 0 dBsm)	m
Time window	409.6	ms
Frame rate	4.9	FPS
FFT point	1024	Point
Overlap size	1023	Point

**Table 2:** Comparative experiments’ results for five different learning models.

Model	Training Time (Minutes)	Recall	Precision	F1	Accuracy
GoogleNet	2.54	0.91	0.92	0.91	0.96
ResNet	1.52	0.89	0.90	0.89	0.96
VGG	12.4	0.95	0.95	0.95	0.98
AlexNet	1.24	0.92	0.93	0.92	0.97
PCA-SVM	2.38	0.92	0.94	0.92	0.97

**Table 3:** Training results based on AlexNet deep learning model: training accuracy and loss.

Case	Training Accuracy	Loss
Case # 1	96.0	0.14
Case # 2	97.0	0.23
Case # 3	96.7	0.14
Case # 4	85.7	0.53

**Table 4:** Probabilistic performance details of radar sensor’s foot gesture recognition according to original and reconstructed radar images.

Compression	Gesture	Recall	Precision	F1
Original	Kicking	0.97	0.9798	0.9749
	Swing	0.96	0.9412	0.9505
	Sliding	0.94	0.9216	0.9307
	Tapping	0.98	1	0.9899
	Object	0.97	0.9798	0.9749
90	Kicking	0.99	1	0.9950
	Swing	0.92	0.9684	0.9436
	Sliding	0.98	0.8909	0.9333
	Tapping	0.97	1	0.9848
	Object	0.97	0.9798	0.9749
95	Kicking	0.98	1	0.9899
	Swing	0.77	0.9747	0.8603
	Sliding	0.9	0.7949	0.8571
	Tapping	0.98	0.9515	0.9655
	Object	0.97	0.9418	0.9557
99	Kicking	0.97	0.9898	0.9798
	Swing	0.84	0.8571	0.8485
	Sliding	0.85	0.7798	0.8134
	Tapping	0.95	0.9314	0.9406
	Object	0.91	0.9785	0.9430
